# From Revolutionary to Stakeholder: Looking at Identity Discourses to Understand the 2016 Short-term Change in China’s North Korea Policy

**DOI:** 10.1007/s11366-023-09847-1

**Published:** 2023-02-14

**Authors:** Nicholas Olczak

**Affiliations:** Department of Economic History and International Relations, Stockholm University, Stockholm, Sweden

**Keywords:** China, Foreign Policy, North Korea, Discourse, Computer-Assisted Text Analysis

## Abstract

During 2016 China’s policies towards North Korea appeared to undergo considerable short-term change, increasingly distancing itself from its neighbour and instead supporting the international community’s response. Existing research has focused on long-term policy change and given little importance to short-term changes in policy, or has drawn on realist and constructivist theories which expect consistency and struggle to account for these changes. This article took an identity discourse approach to understanding the 2016 short-term changes in China’s North Korea policy. It used quantitative computer assisted text analysis methods to measure changes in the dominance of different identity discourses related to North Korea that are produced on the Chinese Internet. It found that around 2015–2016, a previously more dominant “revolutionary” identity discourse lost dominance to a “stakeholder” identity discourse. The article argues that this change made possible the shift in approach to North Korea at the start of 2016 and indicates ways the short-term policy changes at this time may contribute to longer-term change in China’s behaviour.

## Introduction

During 2016 China’s policy towards North Korea underwent considerable change. After North Korea’s fourth nuclear test, China worked with the United States (US) to draft Resolution 2270, and then supported the United Nations Security Council in adopting this resolution which contained the strongest ever sanctions against North Korea to date [1]. There followed a period when China gave extensive support to the international community and showed an unprecedented willingness to cooperate with the US in confronting North Korea [2]. In 2017, China suspended coal imports from North Korea [3, 4] and its state-owned banks restricted the financial activities of North Korean entities.

These changes in China’s North Korea policy present a puzzle for research applying mainstream international relations theories to explain. Strands of realist theory see China’s policies regarding North Korea (and other issues) as principally driven by concern for security in a dangerous and anarchic international system and by its great power rivalry with the US [5]. They expect policies to change only with a major shift in the material balance of power in the international system and a resultant change in the threat to China. In this case no such material power shift appears to have occurred. Although North Korea was becoming more threatening, the US remained the biggest competitor to China’s interests and by far the larger threat in a way which would suggest a continuation of pre-existing security policies. In fact, with its ongoing “pivot to Asia” and proposal to install a THAAD missile system in South Korea, US power projection and security competition was increasing rather than declining.

Mainstream constructivist approaches view China’s North Korea policies as driven by norms and ideas [6], expecting policy change to be the result of large-scale changes in the norms underpinning relations between the two states. However, for the 2016 policy changes it is not apparent that such a shift has occurred. These changes also occurred in a short timeframe. While a shift in norms would suggest a gradual change in the relationship, here China moved from support and refusal to condemn North Korean actions in 2010 [7] to a different approach six years later. This suggests the reason for these policy shifts is something other than gradually evolving norms.

Other studies take a theoretically eclectic approach to explaining such changes in China’s North Korea policy [8]. This views the changes as the result of both a changing level of material threat from North Korea (with its frequent missile testing and nuclear development) and also a more ideational change in how this threat was being understood by Chinese policymakers, for example recognising the particular threat to China’s north-eastern regions. A problem with such an explanation is that even though North Korea’s activity was posing an increased threat to China (and particularly the northeast) during 2015 and 2016, this was overshadowed by the broader security threat presented by the US expanding its power. North Korea’s increased activity could only be seen as a greater threat than the longstanding security threat from the US if there was a changed understanding of what is being threatened, or a change in China’s conception of its identity. If China understands itself as being economically and politically tied into the international community, then North Korea would present a significant threat to this identity.

The puzzling changes in China’s policies towards North Korea in 2016, which approaches drawing on mainstream IR theories do not appear to be able to completely account for, motivate this study’s research question: *How can we understand the changes in China’s North Korea policy shown at the start of 2016 and the considerable cooperation with the international community in the two years after this?*

I define policy changes such as those in 2016 as short-term changes, in contrast to more long-term evolution, because they take place within five years of very different policies. Existing research, focused on the longer-term evolution of China’s overall behaviour, has paid little attention to these issue specific short-term changes. I argue that these changes are important to understand because they are connected with longer-term change in China’s foreign policy. Short-term changes, even if they are sometimes aborted or reversed, set the context for subsequent changes. An identity-based understanding of these changes can help uncover this role.

Analysis of these changes using mainstream IR theory is limited in its ability to explain them. This article instead uses critical constructivist IR theory to understand the changes that China’s North Korea policy underwent in 2016. Critical constructivist theory, drawing on poststructuralism, holds that discursively constructed identity discourses make possible (or thinkable or natural) foreign policies adopted by states at different times [9, 10]. Importantly, this theory does not argue for a causal link between discourses and policies, or for mechanisms/processes translating discourse into policy. The poststructuralist ontology rejects the separation of identity discourse and foreign policy and the notion that the former “causes” the latter. Instead, identity discourses that are co-produced by members of a social group are seen to make possible foreign policy which is also a part of the discourse [9].

A change in dominant identity discourse enables a change in policy, where in using the term enable I mean that it makes possible particular policies, while earlier these policies were not possible. This article provides evidence of change in the dominant identity discourse in relation to North Korea that is being co-produced by the state and Chinese public and argues this change in discourse made possible the shift in policy seen at the start of 2016.

The article makes three contributions: The first contribution is to the literature studying China’s North Korea policy. The article provides an identity-based understanding of the policy change which occurred during 2016, a type of change other studies have struggled to explain. This is an important policy change, and needs to be understood, because it was a considerable shift towards supporting the international community. The second contribution is to show how studying identity discourse can provide understanding of short-term foreign policy change. Existing literature has neglected these changes in specific issue areas, focusing on the broader change in China’s behaviour. It is important to understand these issue-specific short-term policy changes because of how they play a role in longer-term evolution of foreign policy by creating the context for subsequent changes. A third contribution is methodological, demonstrating a method for quantitatively analysing changes in identity discourses constructed in large numbers of texts.

The article proceeds in the following way: First, it provides a description of changes in China’s North Korea policies over the past two decades and a review of existing studies of these policies, pointing to the limitations of these approaches and motivating an identity-based approach. Next, it describes the poststructuralist understanding of identity formation and the critical constructivist IR theory employed to understand the 2016 changes in China’s North Korea policy. It then presents findings from quantitative analysis examining the shifting dominance of different identity discourses articulated by Chinese society. It shows how changes in the dominant identity discourse constructed by Chinese society made possible the shift in China’s North Korea policy in 2016. Finally, the article discusses how understanding the 2016 short-term change can contribute to a broader understanding of China’s evolving North Korea policy.

## Understanding Short-term Change in China’s North Korea Policy

During 2016, China’s North Korea policy appeared to undergo considerable short-term change. This followed a long succession of short-term changes to China’s policies regarding North Korea. Overall, the China-North Korea relationship has been described as an uncomfortable partnership, which emerged after WW2, between two revolutionary states resisting US imperialism [11, 12]. It has been shown how throughout the Cold War, bilateral relations oscillated between amicability and distrust as a result of changing attitudes in both states [13]. China’s interests and behaviour towards its neighbour became increasingly complicated and contradictory as it reintegrated into the global economy in the 1980s [11]. In the two decades preceding the 2016 policy change, these contradictory interests produced considerable short-term change in China’s North Korea policies.

In the 2000s, China initially showed some distance from North Korea and support for the international community’s efforts to rein it in, particularly as leader of the Six Party Talks [11]. However, in the later 2000s it generally supported North Korea and only minimally cooperated with the international community’s attempts to challenge it [7]. China did not help punish North Korea when it shelled South Korea’s Yeonpyeong Island and sunk the South Korean warship Cheonan in 2010 [7]. Although it supported sanctions after the 2006 and 2009 nuclear tests, this was only after pushing for them to be watered down [14]. The 2010s then saw several shifts in China’s policy. After North Korea’s third nuclear test in 2013, China shifted to increased cooperation on UN sanctions [14].

Existing research has often overlooked these short-term changes in China’s North Korea policy because it has been primarily focused on China’s longer-term evolution and the question of whether it will rise peacefully. There has been a focus on overall foreign policy trends rather than those in relation to specific issues such as North Korea and a lack of research using IR theory to explain these issue-specific short-term changes in policy. It is important to understand these short-term changes for two main reasons. They can be critical junctures which contribute to the evolution of China’s foreign policy overall, and, even when reversed, they still contribute to the context in which future policy is made and play a role in longer-term evolution of China’s behaviour. For example, China’s show of support for the international community and leadership role in the Six Party Talks at the start of the 2000s has continued to be part of its identity [15].

Where research has examined China’s North Korea policy, it has adopted a range of theoretical approaches which often expect consistency and so poorly account for short-term policy changes. Many studies use realist theory to explain China’s policies towards North Korea [7, 16–19], viewing these as driven by China’s concern for security in a dangerous and anarchic international system and also by its great power rivalry with the US [5]. For defensive realists, these security concerns make China seek to maintain a status quo and prevent spirals of tension. This makes it balance actions supporting North Korea with those supporting the international community in restraining aggressive behaviour [7, 18]. These accounts do not explain the times when China has changed policy and shown a willingness to work with the international community. If China’s main aim was to avoid provocation, then its support for such actions would likely have been more limited.

Other studies drawing on realist theory argue that China’s policies regarding North Korea are driven by its security competition with the US and the balance of power between these two states. They argue China’s security concerns lead it to consistently provide support to North Korea in order to maintain it as a “buffer zone” against external threats, particularly from the US [16, 17]. This might account for the longer-term support China has shown to North Korea (including during the Korean War 1950–1953). However, the theory tends to expect continuity of the “buffer zone” policy and so does not explain times when China distanced itself from and opposed North Korea. Realists would expect such changes to be accompanied by a shift in the balance of power, when no such change appears to have occurred. The US continues to present the main security threat to China and a reason why, on these terms, China should maintain consistent support for North Korea. The 2016 change in China’s North Korea policy occurred at a time when the US was increasing its power presence in the region, through its so-called “pivot to Asia” and deployment of the THAAD missile systems [20].

There have also been studies taking constructivist approaches and arguing that identity and ideas are behind China’s policies regarding North Korea [6, 21, 22]. These studies tend to share with the realist approaches the expectation of consistency in China’s North Korea policies and so struggle to account for short-term changes. Easley and Park (2016), for example, adopt a norm constructivist approach and argue that China’s policies regarding North Korea are driven by four main norms (including the need for stability, a siege mentality, and ideas of Confucian reciprocity) which guide its approach to regional relations. They explain shifts in China’s policy, showing this is the result of changes in the dominance of these four norms. However, they argue that because these norms are so deeply entrenched, change in China’s policy is likely to be quite limited and overall its normative beliefs produce sustained support for North Korea. As such, this account does not explain those times when China has moved away from support of North Korea.

There has been a growing literature pointing to different domestic factors that shape China’s identity and interests regarding North Korea [15, 23–26], arguing that these can explain recent changes in policy. Many of these studies argue that shifts in Chinese public opinion [15, 25], particularly those being expressed on the Chinese Internet [23, 24, 26], are impacting on the policies that China adopts towards North Korea. Although these studies provide arguments for how domestic factors contribute to evolution in China’s North Korea policy, they typically do not theorise how public opinions and policy are linked and have not accounted for specific short-term changes.

Studies have also taken theoretically eclectic approaches to explaining change in China’s North Korea policy. These suggest China’s North Korea policies are driven by a combination of both material and ideational factors [8, 22, 27]. These would explain changes in policy, such as those in 2016, as resulting from both the growing material power and threat of North Korea, as it develops its nuclear capacity and behaves more aggressively, and also the way in which this threat is perceived in China and the calculations it makes based on this. A problem with this explanation is that despite North Korea’s increasing aggression, the US has continued to be the larger security threat to China. We would therefore expect China’s calculations to continue to favour some support of North Korea. North Korea’s increasing threat is also mainly to China’s northeast, rather than the whole nation. Instead of changing calculations in response to shifting material threats, this suggests changes in policy were made possible by a broader change in China’s understanding of a security situation which remained relatively constant. This shift in understanding can best be studied by looking at changes in China’s discursive construction of its identity.

This article differs from this existing research in taking an identity discourse-based approach to understanding short-term change in foreign policy regarding North Korea. It understands different identity discourses as making possible China’s policies regarding North Korea at particular points in time, and so changes in dominant discourse to lie behind shifts in policy. This critical constructivist theory can be used to understand short-term changes in China’s foreign policy, such as the change seen in North Korea policy in 2016. We can examine changes in the dominant identity discourses being articulated by the whole of Chinese society to make sense of these kinds of puzzling changes in foreign policy. The next section briefly reviews how poststructuralists see identity as being discursively constructed in discourse and how critical constructivist theory links identity discourse with foreign policy.

## Identity Discourses and Foreign Policy Change

Poststructuralist theory understands the identity of an individual or an entity such as a state like China to be socially constructed through a discourse which is shared between members of society [28]. Identity is the meaning given to a particular Self (a person, an organization, or, in this article, China). This identity is constituted through language and there is nothing standing outside of those meanings given by language. Therefore, a state, such as China, does not have an additional, objective, material identity outside of the meanings given to it.

For poststructuralists, language does not have a structured set of fixed meanings (hence the prefix “post”). For example, the word “responsible” does not mean one thing which everybody agrees upon, but is given a range of meanings by people in different circumstances. The use of language, or an *articulation*, partially fixes meaning because it deploys specific words in a way that gives them meaning. Somebody might write that “China is showing it is a responsible member of the international community, unlike the United States”, giving meaning to the word “responsible” within use and through deployment of an Other that is different. Because of the lack of fixed meanings, individuals are compelled to continuously re-articulate identities, trying to achieve ontological security or a firm sense of self [29, 30].

When a shared agreement develops between people in society about meaning, this creates an *identity discourse*. An example is neoliberal discourse which attaches a particular meaning to words such as “freedom” [31] or the “China Dream” discourse and its shared understanding of what it means to be a “great power”. Research has also examined China’s diplomatic discourse and the way that, in all its myriad articulations, this presents a shared understanding of China’s rise and what this means for the wider world [32]. These shared discourses form the pre-conditions for articulations about identity made by members of the group [33]. They act like a set of rules about what can be said. To make a statement that did not fit with this dominant identity discourse would be to open yourself up to social ridicule, shaming, or other forms of exclusion. For example, the dominant nationalistic discourse in China means that making statements about Chinese weakness would place you at odds with society. When a particular discourse becomes very dominant or hegemonic, it is accepted as the “common sense” understanding by most of the social group, such that other statements become difficult to make. These dominant discourses have been linked to government legitimacy, with recent research indicating how during the COVID-19 pandemic the governments of Turkey and China both exploited dominant nationalist identity discourses about a positive “Self” and negative Others to bolster support for their rule [34, 35]. This shows how governments can be invested in reproducing those dominant identity discourses which resonate with the wider public in order to ensure continued legitimacy.

The way meanings can never be entirely fixed, and discourses must be continuously re-articulated, is central to both continuity and change in identity. When articulating an identity, people draw upon pre-existing linguistic materials and repeat previous discourses. They might also draw on broader identity discourses. In articulating an identity for China, people might invoke a wider “rejuvenation” discourse [36] or they might reference broader ideas about China’s role as a “responsible stakeholder” [37]. This means that particular identity discourses tend to persist over time or to resurface after latent periods. Analysis of how individuals articulate identity discourses can examine how they both reproduce and change existing discourses.

The dominant identity discourses can also change. Identity change has been much discussed in the literature, particularly the problem with arguing that identity discourses both structure future articulations and can be challenged [33, 38]. To resolve this, studies have proposed a layered model of discourse where the deeper layers are more fixed, while shallower levels see more contestation [33, 39]. Whenever somebody articulates an identity they can change or contest dominant discourse. For example, a Chinese text might give new meaning to “patriotism” [40]. Research has argued this discursive change is often carried out by “identity entrepreneurs” [38, p.8], or influential individuals involved in the discussion of the topic who advance new meanings. The discussions of China-North Korea relations analysed in this paper show evidence of these kinds of identity entrepreneurs including academics and journalists [41]. Research has also shown that, possibly because of the legitimacy benefits of reproducing dominant discourse that have been discussed above, the articulation of identity discourses by the Chinese state is responsive to broader shifts in public sentiment [42], which could serve as another means for change in the dominant identity discourses shared by state and public.

### Identity and Foreign Policy

Drawing on this poststructuralist theory of identity, critical constructivist IR theory adopts a discourse orientated approach to understanding foreign policy. It holds that dominant identity discourses within society make possible foreign policy [9, 10, 29, 33, 43]. Dominant identity discourses work to delineate policy, making certain courses of action possible, thinkable, or legitimate, and other types of actions almost unthinkable [43]. An example is the “pacifist” identity discourse in Japan, which constructed post-WW2 Japan as a peace loving and non-militaristic society. When dominant, this identity discourse made natural a foreign policy of economic cooperation and minimal military involvement [44]. Similarly, research has shown how dominant identity discourses in Scandinavian states have delineated these countries’ approaches to European integration [33] and how US and Iranian identity discourses about “terrorism” have shaped their policies and contributed to the discord between them [45].

The idea that dominant identity discourses make possible foreign policy also then entails that changes in these discourses enable changes in policy [33, 43, 46]. By enable, I mean that these changes in dominant discourse make particular policies possible, when earlier these policies were not possible. To analyze foreign policy changes, we can therefore look at how the dominant identity discourses that underpin these policies are changing.

To understand short-term changes in China’s North Korea policy, such as those seen in 2016, we might employ this critical constructivist approach and look for the changes in the dominant identity discourses regarding North Korea that made these policies possible. Critical constructivism assumes that where a change in the dominant identity discourse related to the issue is found, this discourse change can be understood to have made possible shifts in foreign policy. This is different from saying that the identity discourse ‘caused’ the change in policy, with poststructuralists seeing foreign policy as itself a part of discourse and so rejecting the notion of causation [9]. Rather than treating the policy shift as something materialized/caused by a change of dominant discourse, this sees the discursive change to make possible the new policy adopted towards an issue such as North Korea, when previously this policy was not possible. Changes to the dominant identity discourses within society can therefore provide a way of understanding short-term policy change.

An objection to this argument would be that, instead of making possible policy changes, the changes in identity discourses are instead a response to policy changes which have already occurred – they are signs of the state justifying new policies it has already adopted. However, this would then require some other reason for the policy change, when, as has been discussed earlier in this article, accounts using material or rational factors to explain the changes have often been unable to do so. In addition, if the discourse change was a result of justificatory statements, the evidence would be different from that seen. We would see a peak in a particular kind of statements only immediately after foreign policy changes are adopted instead of broader changes in the dominance of discourses occurring both before and after policy shifts.

This argument about justification also ascribes considerable agency to state actors, suggesting they are free to make strategically calculated statements to influence general opinions. This disregards the way state actors are themselves embedded in discourses, so that when they produce statements they are also drawing on and reproducing pre-existing discourse shared between members of society. It is unlikely these actors can completely shift societal discourses each time policy changes. Instead, while it is possible state actors sometimes provide justifications of policies retrospectively, they should also be understood as contributing to the ongoing construction of an identity discourse that is shared between members of society and which enables policy.

Where broad changes in the dominant discourse produced by the Chinese state and members of the public are apparent in the period around a particular policy change, this should be seen as enabling the policy change rather than an attempt by the state to justify it. The following section describes the quantitative text analysis methods used to analyze the changes in the dominance of identity discourses underpinning China’s North Korea policy in the period around the 2016 change policy change.

## Analyzing Change in Identity Discourses About North Korea

The article asks whether changes in identity discourse made possible the 2016 change in China’s policy regarding North Korea. I investigated how China’s identity in relation to North Korea was being articulated by state and non-state actors within the discourse community in the period around this policy change. I therefore chose to look at online identity discourses about North Korea over the four-year period 2014 to 2018, which is two years either side of the short-term change focused on in this paper.

The material used consisted of texts produced online by state media and members of the public. Critical constructivists see the identity discourses that underpin foreign policy as constructed by ‘texts’ (which can also include images, films, etc.) produced and shared between members of a discourse community. When analysing these identity discourses and the way in which they enable foreign policies, it is therefore beneficial to look at large numbers of texts being produced. A discourse is formed from a collection of texts in relation to each other, and no individual text can be seen as being completely representative of the discourse [33]. The identity discourses producing China’s foreign policy are constructed in texts by both the Chinese state (including leaders’ speeches, policy documents, state media reports) and Chinese society (including academic articles, non-state media, and online or offline texts from the public). This means that many of the actors which existing literature has presented as engaging in discussions about North Korea (such as the state, academics, and the (online) public) can also be seen as involved with the construction of Chinese identity discourses. This article therefore argues for conceptualising their impact on China’s North Korea policy through their role in producing identity discourses.

It was considered important to look at both texts produced by the Chinese state and those produced by members of the public because of the understanding that these together contribute to the discursive construction of China’s identity. Because the Internet is arguably a key site for the discussion of issues and the discursive formation of China’s identity, this study chose to look at those texts which were being produced by the state and public online. I examined texts produced by the Chinese state media in the online edition of *People’s Daily* and by members of the public on the social media platform *Weibo.* While there are many other types of texts contributing to the formation of Chinese identity discourses, both of these kinds of material can be seen as important parts of the broader societal discourse.

*People’s Daily (Renmin Ribao)* might be seen as the most official of the national state newspapers. It is the mouthpiece of the Central Committee of the Chinese Communist Party (CCP) and is controlled by the Propaganda Department of the Central Committee [47]. Its articles can be taken to represent the views of the Chinese leadership [47]. It can be seen as a good source of the official identity discourse about different foreign policy and domestic issues. The newspaper also reproduces content from other state-owned media organisations such as *Xinhua* and newspapers like *Global Times.* The *People’s Daily* online was launched in 1997, sharing content with the print edition as well as containing many more articles.

*Weibo* is one of the most widely used social media platforms in China. Although there have been government crackdowns, it has still seen its number of monthly users grow to reach over 500 million users in 2020 [48]. I would therefore argue that *Weibo* is an appropriate source for online constructions of identity by the Chinese public. However, it should be seen as representative of public discourse with a number of caveats. Only a portion of Weibo users actively engage in discussions of political issues, although studies have suggested there remains a sizeable community of people talking about topics such as North Korea on the platform [26, 49]. The platform does not represent the entire public, but it can be seen to represent the views of an important group within Chinese society – namely younger, better-educated, and city-dwelling members of the population [50]. The platform is subject to censorship and some posts about North Korea are likely to be removed, but research has shown that censorship is primarily of posts which threaten to lead to offline collective action or are directly critical of the government [51] and that there remains space for relatively open discussion of topics such as North Korea [49].

Articles from the *People’s Daily* website were collected using a search for the keyword *North Korea* 朝鲜 in the site’s archive and then scraping software to collect all the articles listed between the start of 2014 and end 2018. This gave me 6742 articles that were about North Korea. The material from *Weibo* was taken from a database of posts compiled by members of the Journalism and Media Studies Department at Hong Kong University. This consisted of all the posts containing the same keyword from the start of 2014 to the end of 2018 [52]. There were 258,083 posts about North Korea. Because the Hong Kong University data collection was interrupted during 2014, there is not a complete set of data for this year. I therefore used data starting from 1 October 2014 unding provided by Stockholm Unand running through until 31 December 2018.

The way identity discourses are constructed through large numbers of texts shared within a society means it is preferable to analyze all of this material rather than samples. Computer assisted text analysis allowed me to do this in a way not possible with qualitative discourse analysis. China’s identity in relation to North Korea is constructed in texts in two main ways. First, particular words or signifiers are used to describe China itself. A text might contain a statement saying that “China is a great nation” for example. Second, an identity for China is constructed by presenting other states as different from it, ascribing particular labels to these Others in a way that says something about China. A statement might describe how “North Korea behaves recklessly and breaks all the rules of the international system” for example, where the presentation of it as a different Other can also reinforce a construction of China as responsible. In short, these two forms of identity construction mean the focus of analysis should be on the labels which are used alongside the names of states, both the Chinese Self (or “China”) and particular Others which China’s identity is constructed in relation to (“North Korea”, “the US”). Computer assisted text analysis provides useful tools for examining which words are used alongside the names of states.

The *Quanteda* text analysis package [53] was used to produced counts of the number of times each quarter that different words were used within a window of five words either side of the name of a state (such as China, North Korea, and the US). The choice of a five-word window was expected to produce the most meaningful data about the texts. Words that appear directly alongside a country name are often not the ones that convey the meaning. In a phrase like “*I think*China*is the* greatest country” (我觉得中国是最厉害的国家 *wo juede zhongguo shi zui lihai de guojia*) those words in italics that are within a two-word window of the country name are not the most meaningful in terms of identity. Therefore a window of less than three would not capture significant words, while a very large window of more than five words would risk capturing words not really associated with the country name.

In order to support this quantitative analysis, and provide an indication of what terms to focus on, close qualitative discourse analysis of a small sample of articles and posts was also carried out. This analysis provided insight into what kinds of labels were being employed to describe the Chinese Self and North Korean Other during the period, and therefore which words might be relevant to examine during the co-occurrence analysis. For example, it showed that the word “hereditary” was often used to describe the political system of North Korea in a way which presented it as different from China, implying China contrastingly had a form of democracy to select future leaders.

The analysis presented below describes the frequency with which relevant words were used in connection with China, and with North Korea, to investigate changes in China’s discursively constructed identity.

## Analysis

### From Competitor to Responsible Stakeholder: The Changing Presentation of China

The first step of the analysis examined how China’s identity was being constructed through words used in connection with “China” itself. There are marked changes in the frequency of particular words used in connection with “China” in both types of texts, and particularly in the *Weibo* posts.

**Fig. 1:**
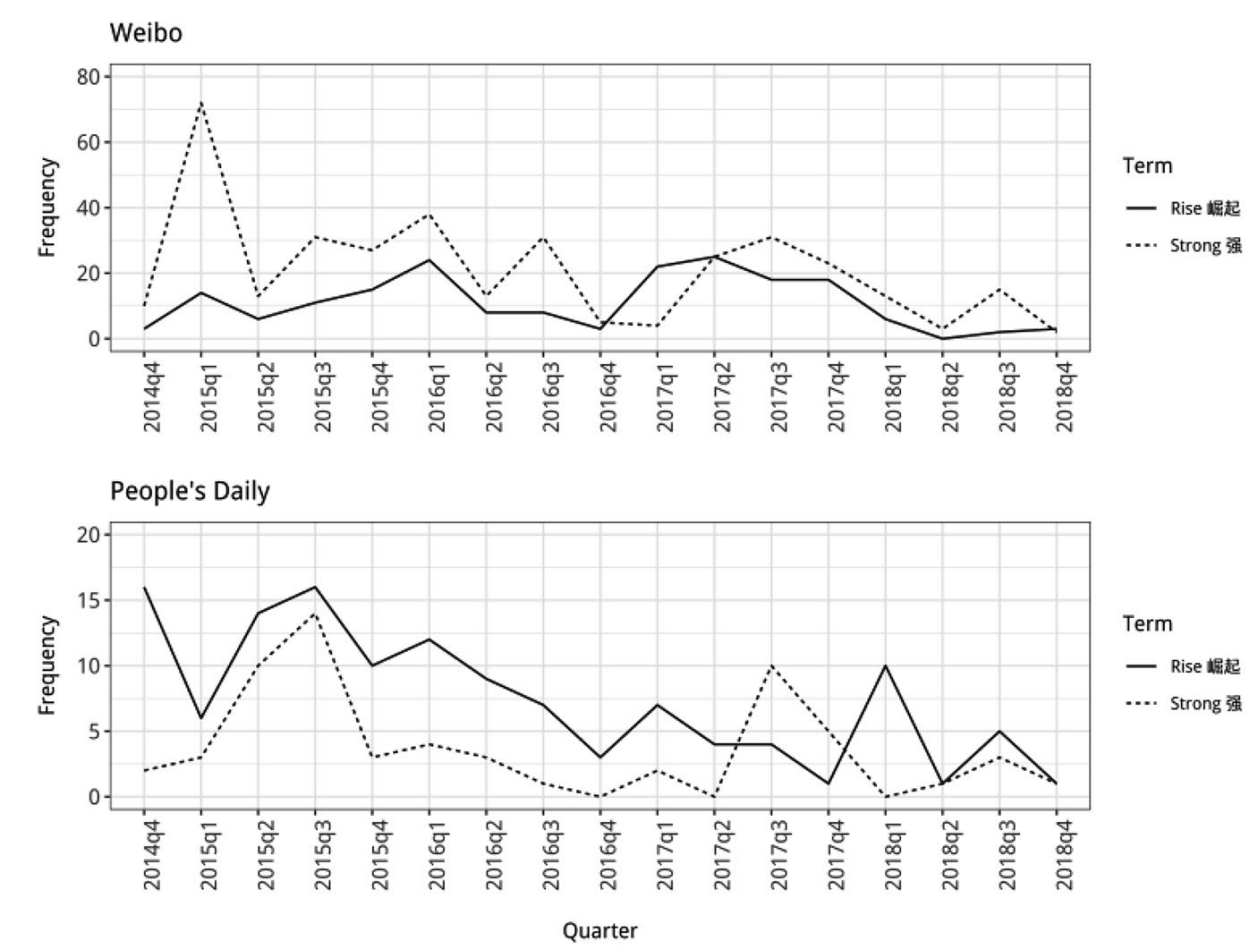
Frequency counts for “rise” and “strong” used alongside China (note different y-axis for People’s Daily)

First, there is a decline in the use of words presenting China as in competition with other powers in the international system (Fig. 1). Particularly on *Weibo*, the word “strong” is used frequently in connection with China in discussions of North Korea at the start of 2015 and throughout the year. After this, its use declines for a period during 2016 and through until the first quarter of 2017. The frequency pattern for the use of the word in *People’s Daily* is similar, with it being used less often alongside China from early 2016 onwards. This indicates that, in the discourse about North Korea, there was much less emphasis on the presentation of China as a strong nation, standing up to other powers, during this period.

The term “rise” might also be used as part of articulations of this identity for China. On *Weibo*, it is not used particular frequently in connection with China through the period, but there is still a noticeable decline during 2016 before an increase the following year. The pattern is more pronounced in the *People’s Daily* articles, with a marked decline in the use of the word “rise” alongside China from the start of 2016 and through until the start of 2018. Overall, this again suggests that there was less emphasis in the discourse about North Korea on China’s increasing material strength.

**Fig. 2:**
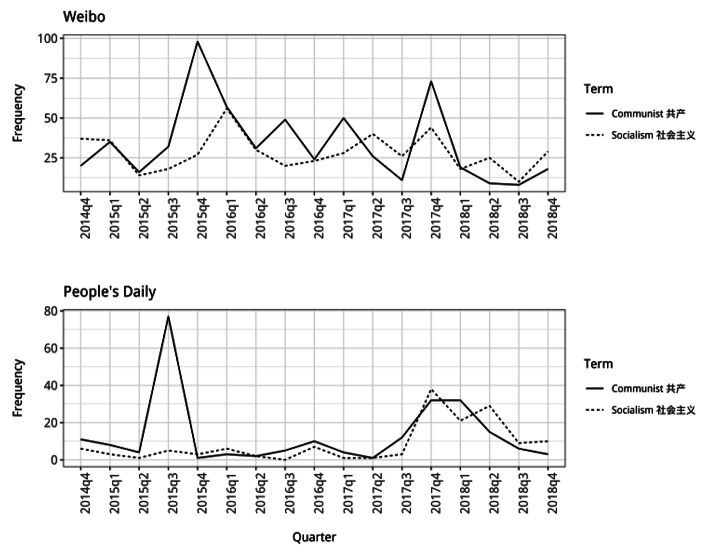
Frequency counts for “communist” and “socialism” used alongside China

The frequency patterns for “communist” and “socialism” alongside China (Fig. 2) somewhat reflect these changes, but offer less conclusive findings. It appears “communist” was used slightly less frequently alongside China between 2016 and 2017, but only relative to its increased use at the end of 2015 (likely related to the anniversary of WW2). While it might be argued there was a slight decline in China’s presentation as a communist nation during 2016 and 2017, the results more suggest a sustained presentation of China as communist. There is similarly little change in the use of “socialism” alongside China on *Weibo*^1^. At the end of 2017 and start of 2018, there is a rise in use of “communist” and “socialism” alongside China on *Weibo* and in *People’s Daily*, with a concurrent rise in “socialism” only in *People’s Daily*. This might indicate a return to the presentation of China in ideological and competitive terms.

**Fig. 3:**
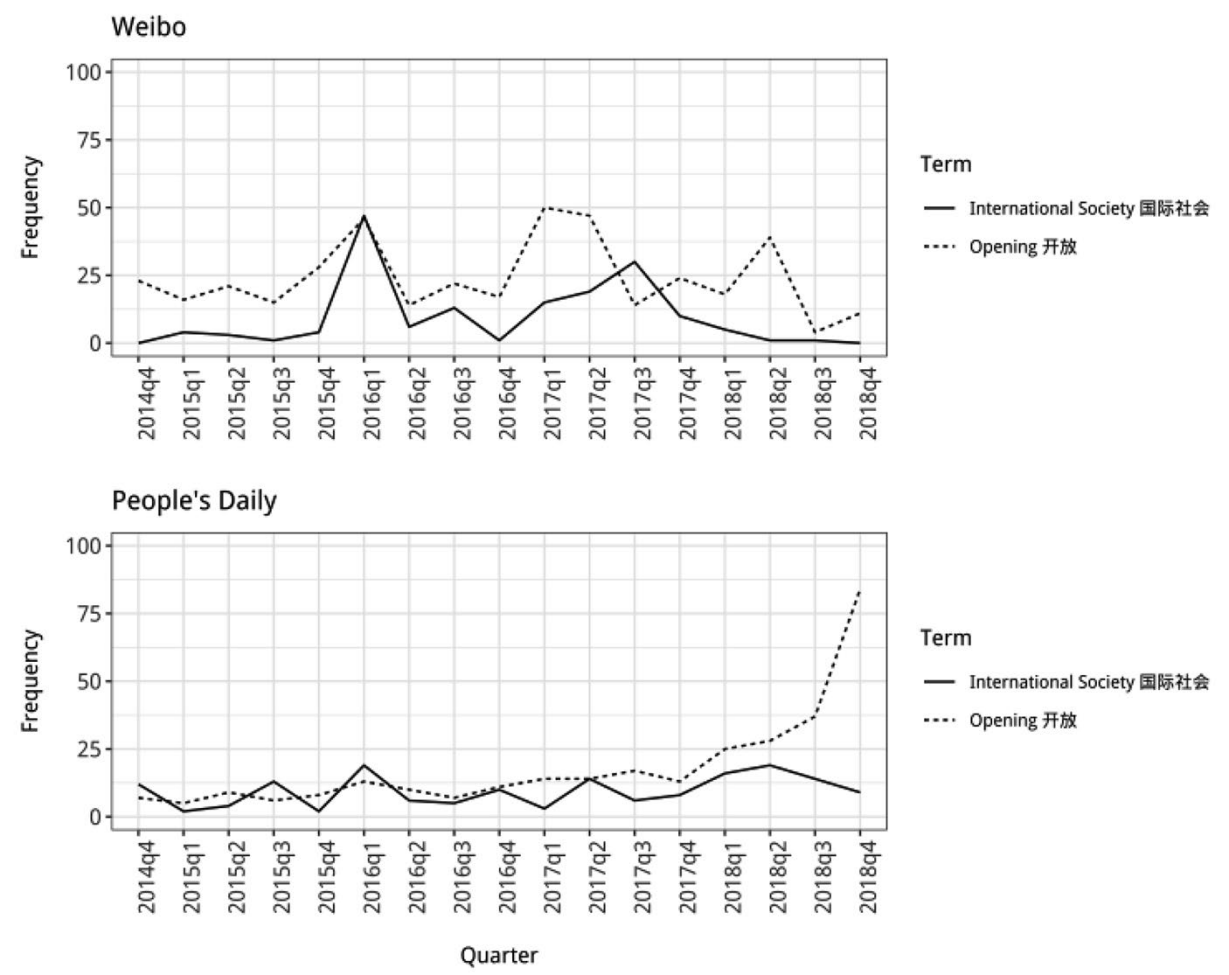
Frequency counts for “opening” and “international society” used with China

Second, from 2016 onwards there appears to be a rise in frequency of use of words alongside China that present it as cooperating/engaging with the rest of the world, particularly in the *Weibo* posts (Fig. 3). In these posts, the frequency of the use of the word “opening” alongside China (part of the idea of China’s reform and opening where it began to engage in greater cooperation with the world) is relatively low until the end of 2015, but then in certain periods it is much higher. In the *People’s Daily* articles, there is also a gradual increase in the use of the word alongside China starting from around the beginning of 2016. This suggests that in discussions of North Korea, from the start of 2016 onwards, there was greater presentation of China as a nation that had undergone reform and opening to integrate into the world.

More striking is the change in the frequency of the word “international society” alongside China in both types of material. In the *Weibo* posts, there is a large rise in the use of this word with China during the first quarter of 2016 and then usage remains high for most of the following two years. In the *People’s Daily* articles, mentions of international society alongside China are also more frequent during this period. This indicates that in discussions of North Korea, during 2016 and 2017, China was being discussed in relation to international society, likely being presented more as a part of the international community. Altogether, the findings for these two words show that between early 2016 and the start of 2018, discourses constructing China’s identity placed greater emphasis on its cooperation with the rest of the world and its role within the international community.

**Fig. 4:**
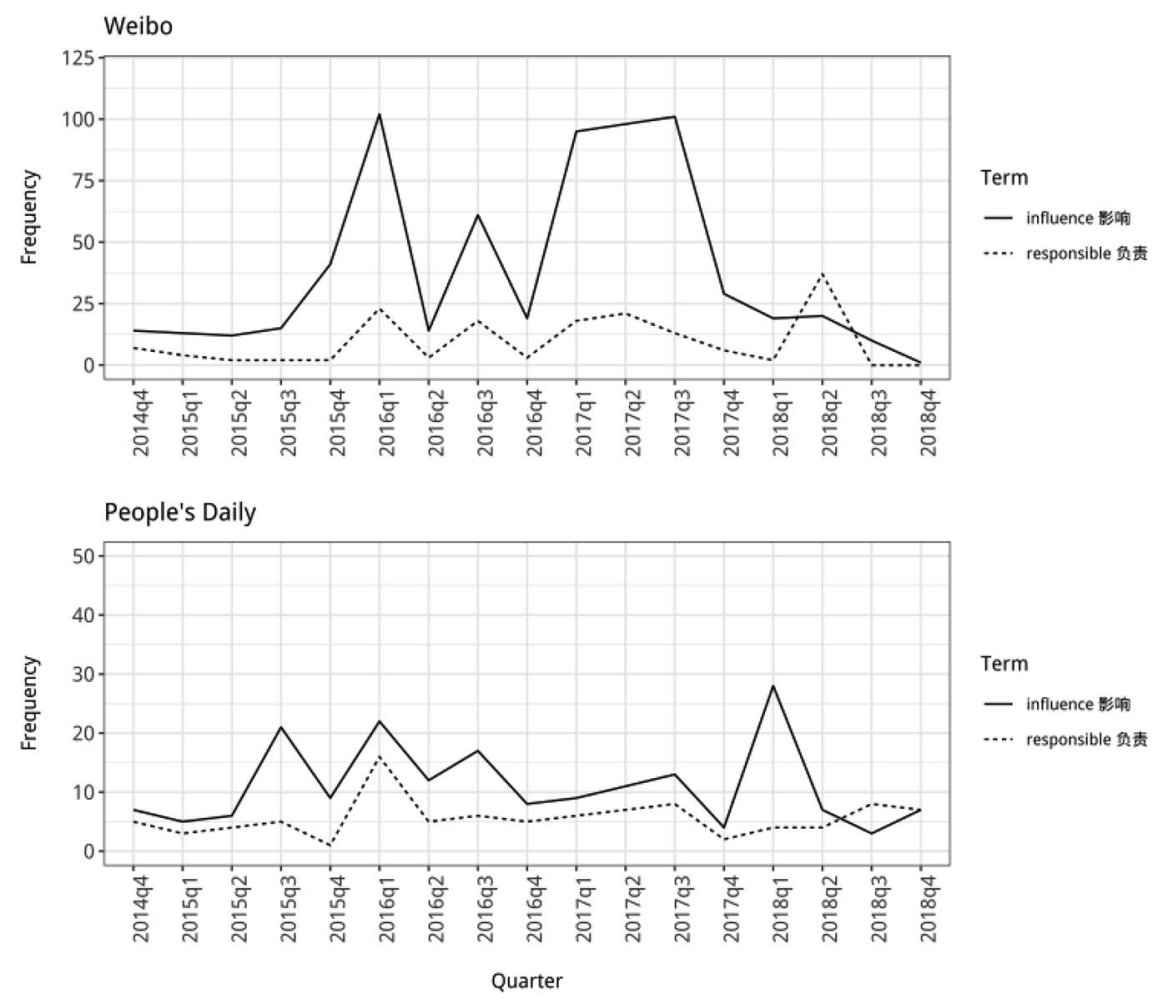
Frequency counts for the words “influence” and “responsible” used with China (note different y-axis scales)

In parallel with this, we also see changes in the frequency of use of particular words describing how China acted in the world during this period (Fig. 4). Alongside increasingly presenting China as a part of international society in the ways discussed above, the discourses also appear to increasingly present it as playing a role, and contributing to, this society.

In both the *Weibo* posts and the *People’s Daily* articles, there is a marked increase in the frequency with which the word “responsible” is used alongside China after the start of 2016. This indicates that China was being presented as a responsible actor in the world during this period. There is a particular rise in the frequency of the use of the word at the start of 2016, which coincided with China’s cooperation with the international community in response to North Korea’s fourth nuclear test. There is a similar pattern for the frequency of use of the word “influence” alongside China, with a marked increase in frequency after the start of 2016 in both types of material. This suggests that in Chinese media discussions of North Korea, China was being presented as an influential actor within international society to a much greater extent during this period than it had been before.

### From Closeness to Difference: Changing Presentations of North Korea

In the second step, the analysis examined the ways in which the discourses presented the identity of North Korea, as the main “Other” in relation to which China’s identity was being constructed. There is a noticeable change in how North Korea is presented during the period being analyzed, with a decline in use of words presenting it as more similar to China and an increase in the use of words that present it as being different. Particularly in the *Weibo* posts, labels expressing North Korea’s closeness with China appear to be used less frequently from the middle of 2015 onwards (Fig. 4). The phrase “wartime friend”, for example, is used more at the start of 2015 and then use declines apart from a slight rise at the end of the same year which might be due to intense discussion at this time. The phrase is hardly used at all in the *People’s Daily* articles, which likely reflects the different kind of language used by state media.

**Fig. 5:**
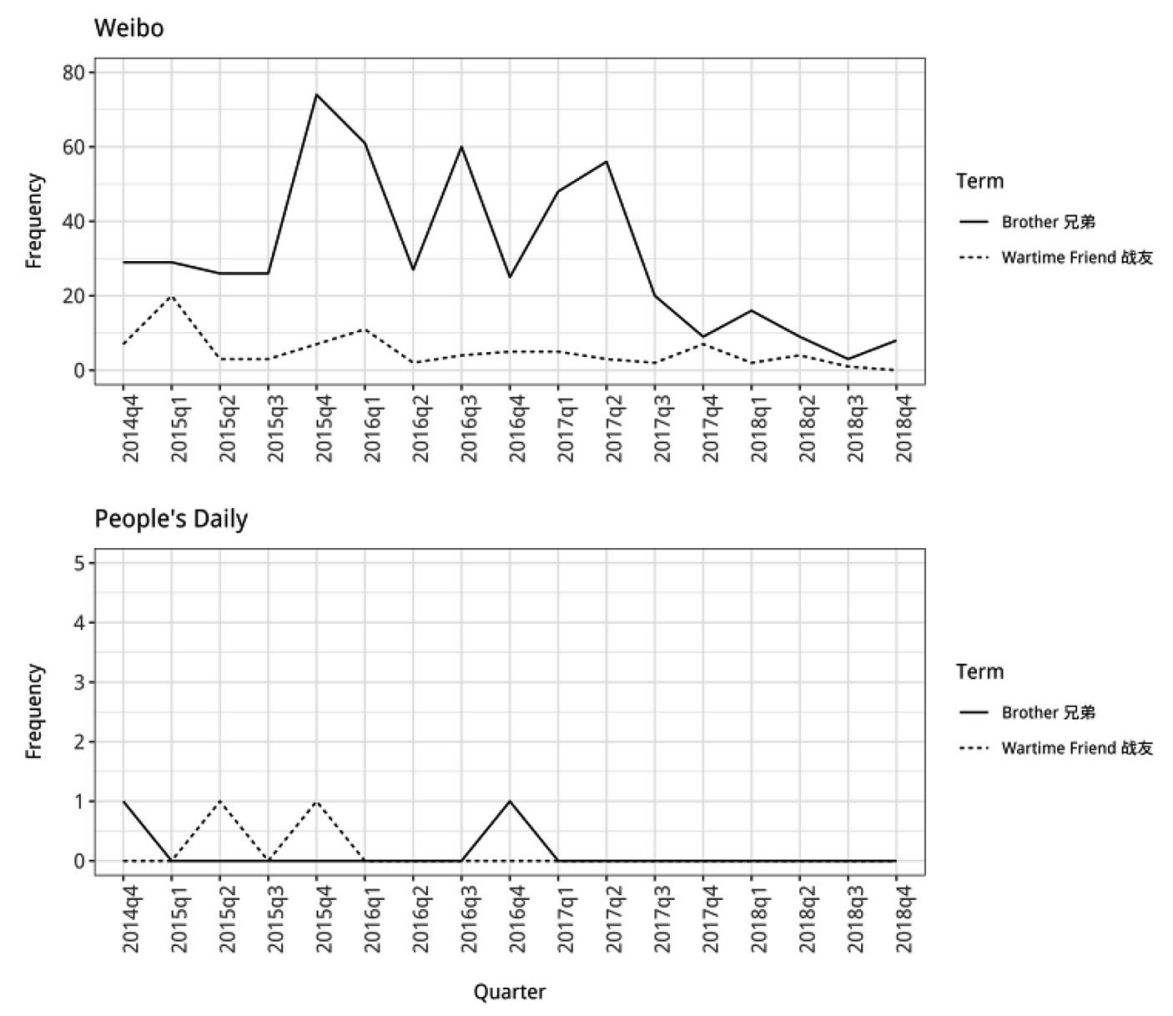
Frequency counts for “brother” and “wartime friend” used alongside North Korea (note different y-axis scales)

Research and my preliminary qualitative discourse analysis indicated that “brother” was another label applied to North Korea to express its closeness and similarity with China [54]. The results do suggest an overall decline in the frequency that this word is used alongside North Korea in the *Weibo* posts, however this is less clear. There are also several points where the term continues to be used extensively. These may reflect times when relations with North Korea were being more intensely discussed (such as around the times of its nuclear tests) and participants in these discussions continued to use the label as they debated China’s relations with its neighbor.

**Fig. 6:**
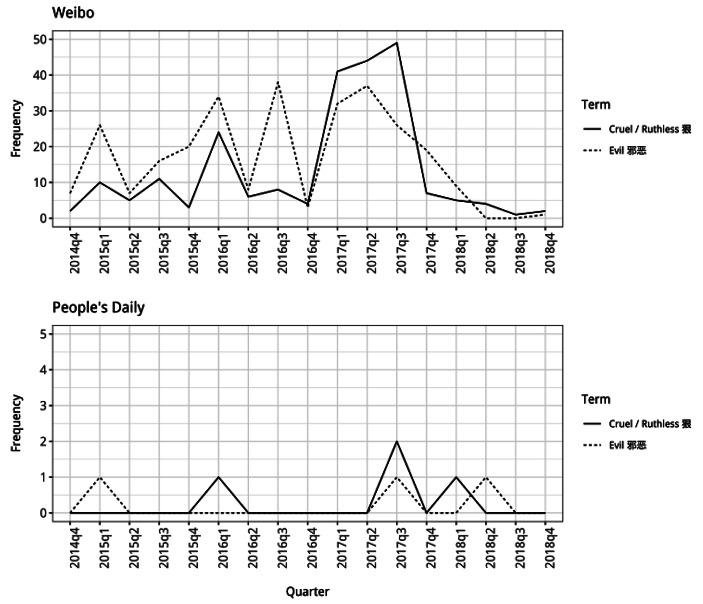
Frequency counts for the words “cruel” and “evil” used alongside North Korea (note different y-axis scales)

The change in the use of words suggesting North Korea’s difference from China is much more distinct, particularly in the *Weibo* posts. Here there is a large increase in the frequency that words suggesting difference are used alongside North Korea from the start of 2016 onwards, with these words being used extensively at periods after this (Figs. 5, 6 and 7). For example, use of the word “evil” alongside North Korea, increased considerably in the first quarter of 2016 and is high in the third quarter of the same year, before being consistently high through 2017. This indicates that the public discussing North Korea on *Weibo* were increasingly viewing it as acting in negative ways during this period, seeing it as very different to China which was being presented as responsible and good. The frequency of use of the word “cruel” alongside North Korea shows a somewhat similar pattern, with much greater use at the start of 2016 and during 2017, although this increased usage is not quite as sustained. Neither of these words are used much in the *People’s Daily* articles which may be due to the fact that they are too emotionally loaded words for inclusion in state media articles, which tend to use more neutral and diplomatic language.

In the *Weibo* posts, the words “dangerous” and “threatening” are also used much more frequently alongside North Korea after the start of 2016 (Fig. 7a and b). The frequency patterns for the two words are similar, with increased use at the start and middle of 2016 and then sustained high use of the words throughout 2017. This suggests that in the discussions taking place on *Weibo* at this time, members of the Chinese public were increasingly presenting North Korea as being very different to China because of the ways in which it poses a danger to it. Although the words are used much less frequently in the *People’s Daily* articles, their patterns are quite similar to those for *Weibo*. From the start of 2016 onwards, the state media articles also used the word “threatening” alongside North Korea much more frequently than before.

**Fig. 7a:**
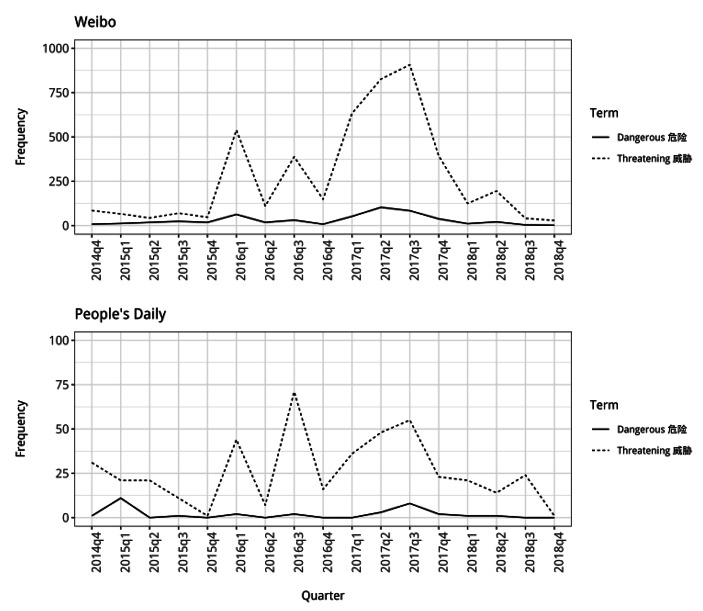
Frequency counts for “dangerous” and “threatening” used alongside North Korea (note different y-axis scales)

**Fig. 7b:**
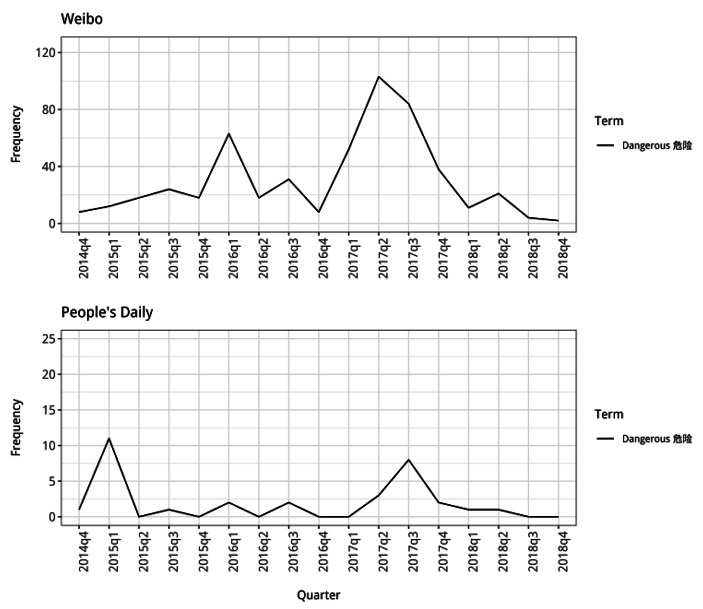
Frequency count for “dangerous” used alongside North Korea (note different y-axis scales)

**Fig. 8:**
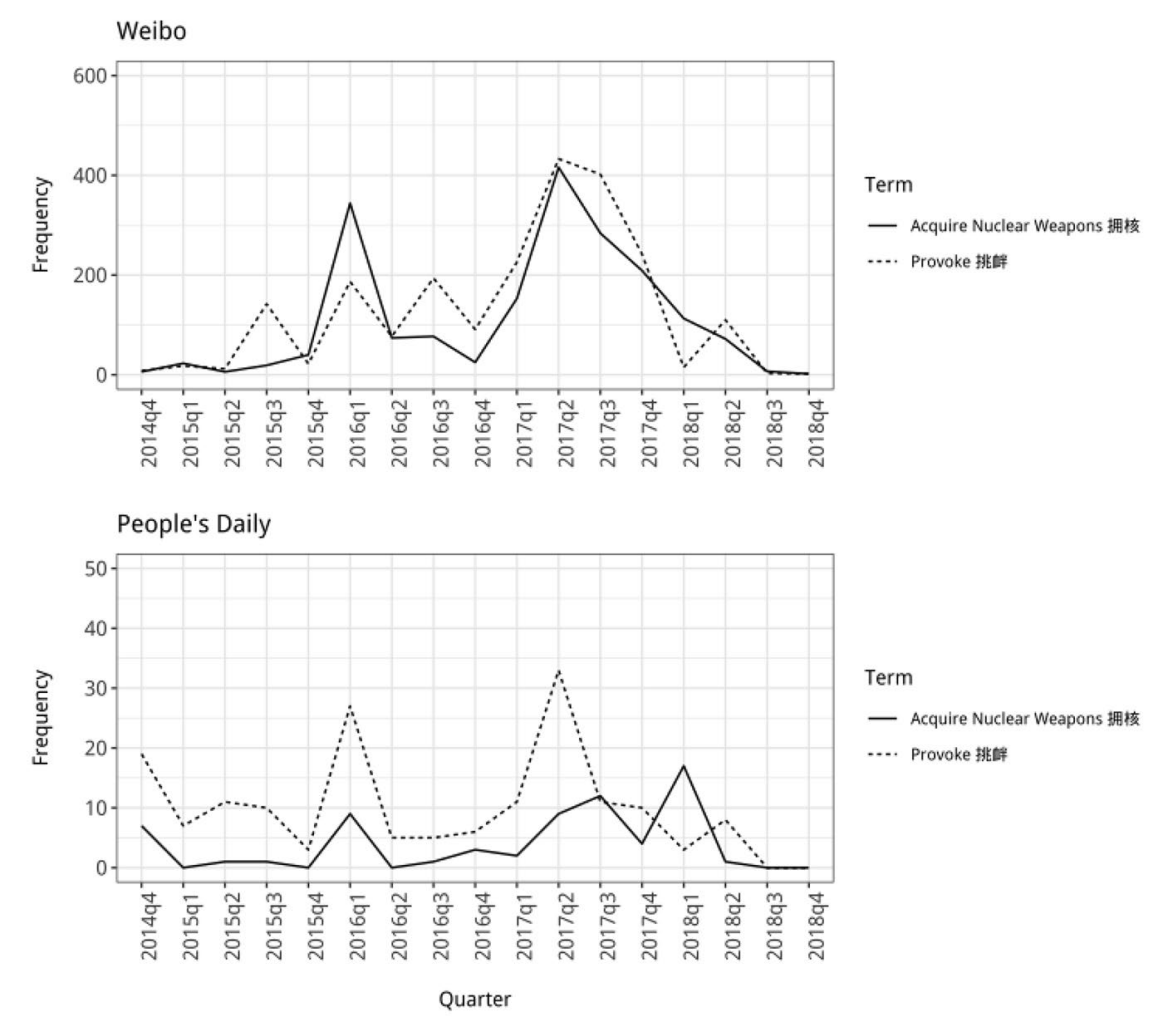
Frequency of use of the phrases “acquire nuclear weapons” and “provoke” alongside North Korea (note different y-axis scales)

Alongside this use of labels to present North Korea as different from China, the texts also describe what North Korea does as a way of presenting it as acting differently. In both the *Weibo* posts and the *People’s Daily* articles, from the start of 2016 there is a substantial increase in the attribution of actions to North Korea that make it different from China (Fig. 8). In the first quarter of 2016, both types of material frequently present North Korea as different from China because it is “acquiring nuclear weapons” (which goes against China’s interests). Although the use of this verb phrase drops during 2016, after this in 2017 the phrase is once again frequently used through the entire year. A similar frequency pattern is present for the use of the word “provoke” alongside North Korea in both types of material. There is much higher usage of the word alongside North Korea from the end of 2015 onwards.

Altogether the increased frequency of use of these two words alongside North Korea indicates that China’s neighbor was increasingly being seen as a state that acted differently from China and challenged its interests. From the start of 2016 onwards, there is a marked decline in the use of words suggesting similarity between China and North Korea and a corresponding rise in the use of words suggesting difference between the two states.

## Discussion

The aim of this analysis was to understand puzzling short-term changes in China’s North Korea policy, particularly those which occurred during 2016. These have been poorly explained by other IR theories which typically expect China to be more consistent in its approach to North Korea, so there is merit to considering this discourse-based account of such puzzling policy shifts.

The analysis shows that, around the start of 2016 when China’s foreign policy underwent significant short-term changes, its identity in relation to North Korea, as constructed in social discourses, also underwent a number of changes. I argue that this change in the identity discourses that were dominant made possible the change in North Korea policy. As elaborated in the theory section, this is not a proposal that the changes in discursively constructed identity shown in the analysis *caused* changes in policy. It is an argument that these changes in dominant societal discourses made possible policy regarding North Korea which previously was not possible. A full account of the 2016 policy change is as follows:

From the middle of 2015 there was a change in the dominant identity discourse underpinning China’s North Korea policy, particularly as this was articulated by members of the public in *Weibo* posts. This involved a change in the way in which China itself was being presented, with less emphasis on its competition with other states and instead the presentation of it as integrated into and cooperating with the international community. The identity discourse that became dominant presented China as a “responsible” member of international society which had considerable “influence” in the world. Alongside this change in the presentation of the Chinese Self, the shift in dominant discourse also involved significant changes in the way in which North Korea was presented in relation to China. There was a decline in the presentation of North Korea as similar to China, with the labelling of it as a “wartime friend” and “brother”, and an increase in the use of words presenting it as being different and threatening. From the end of 2015 onwards, it is particularly noticeable how much more members of the public engaging in discussions on *Weibo* present North Korea as being an Other which is very different to the Chinese Self, describing it as being “evil” and “cruel” and acting in ways which contradict China’s interests.

Altogether, this change in the dominant identity discourse can be described as a change from what I have labelled as the “revolutionary” discourse, where China is described as similar to North Korea and in competition with other great powers, to the “stakeholder” identity discourse, where it is described as integrated into the international community and very different from North Korea. These discourses should not be seen as being entirely new to the period under analysis. Instead, in articulating the “revolutionary” discourse at the start of the period, people were likely drawing on elements of much older, entrenched identity discourses about relations with North Korea, dating back to the Mao-era post WW2. Similarly, in their articulations of the “stakeholder” discourse, people were also drawing on a wider discourse about China’s role in international society which had emerged in the 2000s. In their re-articulation of this responsible stakeholder discourse and connection of it specifically with the issue of North Korea, the Chinese state media and members of the public made this identity discourse more dominant.

The rise to dominance of the “stakeholder” identity discourse, and the decline in dominance of the previously stronger “revolutionary” discourse, made possible the short-term changes in China’s North Korea policy that were seen at the start of 2016 and continued during the two years after this. Previously, when the “revolutionary” identity discourse was still more dominant, it made natural or necessary continued support for North Korea because of the shared identity of the two states in opposition to, and competition with, the West. Once this identity discourse lost its force, and the “stakeholder” discourse became more dominant within Chinese society, this made more natural or legitimate China’s cooperation with international society to do something about the increasingly different and threatening North Korean regime.

Thus, the dominance of the “stakeholder” discourse made possible the policy shift in March 2016 which saw China cooperating extensively with the US and drafting punitive United Nations sanctions in response to North Korea’s fourth nuclear test. It also enabled the subsequent policy decisions which saw China suspending coal imports from North Korea during 2017 and freezing the financial assets of North Korean entities in its state-owned banks.

The change in the dominant discourse behind China’s North Korea policy at this time occurred because more of the texts produced by members of the online Chinese public and the state media were articulating versions of a “stakeholder” identity for China. While it is beyond the scope of this article to fully investigate why there was this change in dominant discourse, several reasons might be proposed. One is that there were a number of “identity entrepreneurs” at the time including influential academics and journalists who began to articulate a stakeholder identity for China which may have been picked up and reproduced by others, helping initiate this change in the discourse [41]. Another reason may be that the specific identity discourses concerning relations with North Korea were interacting with broader identity discourses very dominant at this time. In 2015 and 2016, there was a strong push to present China as a global leader in connection to its recently launched Belt and Road Initiative and also in connection with its participation in different areas of UN activities, such as the 2015 Paris climate change summit. The presence of these broader identity discourses may have helped the stakeholder discourse to become more dominant in North Korea-specific discussions.

It is significant that this change in discursively constructed identity occurred primarily in the articulation of China’s identity in posts being made by the public on *Weibo*, rather than in the state media articles produced by *People’s Daily*. This might suggest that the change in discourse was not only the result of the state media responding to, and seeking to justify, foreign policy changes which had already been made for other reasons. If this had been the case, then we would likely have seen the *People’s Daily* instead leading the changes in the identity discourse being expressed, with its state media articles containing the most frequent expressions of the “stakeholder” discourse. Instead, this identity discourse, and particularly the strongest descriptions of North Korea as a different Other, was most frequently articulated by the public on *Weibo.* While the data in this study is insufficient to draw firm conclusions about whether the public or state media are more influential in producing dominant discourses, this finding indicates that the public at least play a role. It could support studies that argue the discussions taking place domestically amongst members of the public within China impact on its foreign policies regarding the Korean Peninsula [23, 25, 26, 55]. Further research should be carried out to examine the extent of the public’s influence in producing identity discourses that enable foreign policy.

The argument that the 2016 short-term policy changes were made possible by a change in dominant discourse can be considered alongside several alternative explanations (outside of the theoretical accounts discussed earlier). Other explanations might argue that these changes were the result of changes in North Korea’s behavior or were simply the result of the US successfully persuading the Chinese leaders to change track. While it is true that at the time North Korea was becoming increasingly provocative and active in its nuclear testing, this was ongoing for an extended period of time and does not explain these particular policy shifts. The explanation based on US persuasion is also weakened by the fact that during this period, although showing greater willingness to cooperate with the international community, Chinese discourses continued to present the US as a different Other.

The effectiveness of looking at identity discourses to make sense of the short-term changes in North Korea policy during 2016, means that using the same approach may account for other recent policy shifts. In 2018, China appeared to revitalize relations with North Korea and the leaders Xi Jinping and Kim Jong-Un met several times. This may have been made possible through the renewed dominance of a “revolutionary” identity discourse within Chinese society which presented greater similarity between the two countries, set against depictions of the US as a shared Other very different from and threatening to both states in the same way. Examining changes in the dominance of discourses within Chinese society could therefore help to explain how this 2018 policy shift was possible even though it followed on from a period where China had shown considerable support to the international community in confronting North Korea, with a Chinese public discourse that was increasingly hostile towards its northern neighbor. However, a nuanced analysis of this recent warming in China – North Korea relations would also look for persistence of the stakeholder identity discourse and its potential reemergence as dominant.

This article argues it is important to understand short-term policy changes in relation to particular issues, such as North Korea, not only because they can be impactful themselves, but because they may play a role in the longer-term evolution of China’s behavior overall. These changes arguably set the context in which subsequent policy changes are made. An identity discourse-based understanding of the short-term changes can provide insight into how they do this. The stakeholder identity discourse which made possible changes in policy in 2016 may persist and form part of the context in which future policy is made the next time North Korea engages in aggressive actions or carries out nuclear tests. Having taken actions which are an expression of this stakeholder identity in 2016, China creates expectations that it may live up to this identity in the future. Subsequent articulations of China’s identity are likely to reference the policy made at this particular point in time.

## Conclusion

This article asked how we can understand the 2016 short-term change in China’s foreign policy that saw it increasingly distancing itself from North Korea and giving extensive support to the international community. It argued that it is important to understand such short-term changes in policy not only because they are impactful in themselves, but also because of the role they play in the longer-term evolution of China’s behavior by setting the context for subsequent changes.

The 2016 changes in policy can be understood using a critical constructivist approach and analyzing changes in dominant identity discourses. A quantitative analysis of the discursive construction of China’s identity in relation to North Korea by members of the public and the state media between 2014 and 2018 found that a shift in the dominant identity discourse occurred around the time of these changes. There was a decline in the dominance of an older discourse which presented China in opposition to international society and similar to North Korea, and a rise in the dominance of a discourse which presented North Korea as being a different other that was threatening to China. This change in dominant discourse, which was particularly seen in the public discussions taking place on *Weibo*, made possible the policy shift seen at the start of 2016, when China began to demonstrate much more extensive support for the international community’s efforts to confront North Korea’s nuclear development. The newly dominant identity discourse within Chinese society helped to make natural the strong actions which China took as part of efforts to rein in North Korea.

Although the short-term shift in policy has since been partly reversed, and China-North Korea relations have revived since 2018, it is likely that the actions taken during this period and the identity discourses behind them will continue to inform the context in which policies about North Korea are made and so play a role in the longer-term evolution of China’s behavior. As the situation on the Korean Peninsula continues to develop, and different actors within the Chinese state and wider society continue to discuss the issue and try to make sense of their country’s identity and relations with its restive northern neighbor, it can be expected that further shifts in the dominance of different identity discourses will occur making possible more short-term changes in policy. As Chinese identity evolves, previous short-term changes may be referred to as part of the context in which new policies are made.

This article has demonstrated how a critical constructivist approach can be used to understand short-term changes in China’s foreign policy, such as the ones relating to North Korea in 2016, and to uncover the role these changes may play in the longer-term evolution of China’s behavior. Further research into the ongoing shifts in the identity discourses being produced by Chinese society, and the extent that the public contribute to producing discourses, can help us to better understand China’s changing foreign policy in both the short and longer-term.

## Footnotes

1 The qualitative discourse analysis of a smaller sample of texts from across the four years carried out to accompany this article appeared to confirm this. China continued to be presented as a communist state throughout the period, but was increasingly presented as one which practiced a very different form of communism from that of North Korea.
